# Reactive Sulfur Species Interact with Other Signal Molecules in Root Nodule Symbiosis in *Lotus japonicus*

**DOI:** 10.3390/antiox9020145

**Published:** 2020-02-07

**Authors:** Mitsutaka Fukudome, Hazuki Shimada, Nahoko Uchi, Ken-ichi Osuki, Haruka Ishizaki, Ei-ichi Murakami, Masayoshi Kawaguchi, Toshiki Uchiumi

**Affiliations:** 1 Graduate School of Science and Engineering, Kagoshima University, Kagoshima 890-0065, Japan; k2932465@kadai.jp (M.F.); uchi-n@d1.dent.kagoshima-u.ac.jp (N.U.); abamineaba@yahoo.co.jp (K.-i.O.); 2 Department of Chemistry and Bioscience, Kagoshima University, Kagoshima 890-0065, Japan; k9026178@kadai.jp (H.S.); k7051939@kadai.jp (H.I.); 3 Graduate School of Medical and Dental Science, Kagoshima University, Kagoshima 890-0065, Japan; 4 Division of Symbiotic Systems, National Institute for Basic Biology, Okazaki 444-8585, Japan; murakami@gragreen.com (E.-i.M.); masayosi@nibb.ac.jp (M.K.)

**Keywords:** hydrogen sulfide, nitric oxide, nitrogen fixation, reactive oxygen species, reactive sulfur species, symbiosis

## Abstract

Reactive sulfur species (RSS) function as strong antioxidants and are involved in various biological responses in animals and bacteria. Few studies; however, have examined RSS in plants. In the present study, we clarified that RSS are involved in root nodule symbiosis in the model legume *Lotus japonicus*. Polysulfides, a type of RSS, were detected in the roots by using a sulfane sulfur-specific fluorescent probe, SSP4. Supplying the sulfane sulfur donor Na_2_S_3_ to the roots increased the amounts of both polysulfides and hydrogen sulfide (H_2_S) in the roots and simultaneously decreased the amounts of nitric oxide (NO) and reactive oxygen species (ROS). RSS were also detected in infection threads in the root hairs and in infected cells of nodules. Supplying the sulfane sulfur donor significantly increased the numbers of infection threads and nodules. When nodules were immersed in the sulfane sulfur donor, their nitrogenase activity was significantly reduced, without significant changes in the amounts of NO, ROS, and H_2_S. These results suggest that polysulfides interact with signal molecules such as NO, ROS, and H_2_S in root nodule symbiosis in *L. japonicus.* SSP4 and Na_2_S_3_ are useful tools for study of RSS in plants.

## 1. Introduction

Nitric oxide (NO), reactive oxygen species (ROS), and hydrogen sulfide (H_2_S) function as signal molecules in physiological responses, including germination, stomatal regulation, and photosynthesis, and in biotic/abiotic stress responses in plants [1,2,3,4]. Each molecule functions not only independently, but also collaboratively or interactively. NO reacts with ROS to form reactive nitric oxide species such as peroxynitrite, which participates in protein activity by posttranslational modification such as tyrosine nitration [5,6,7]. Both NO and ROS regulate expression of genes related to stress responses in plants via primary metabolism and plant hormone signaling [8,9,10]. H_2_S is involved in regulation of ROS, NO, antioxidant levels, abscisic acid, K^+^ channels, and stomatal movement [11,12,13,14,15,16].

Plants produce NO and ROS in response to infection by microbes [2,5,17,18,19,20], and NO and ROS production is part of the process for establishment of plant–bacteria symbioses [21,22]. In legume–rhizobia symbioses, NO and ROS are detectable and function throughout the symbiotic process, including development of nodules and symbiotic nitrogen fixation [23,24]. However, the role of NO in the symbiosis is still not fully understood, because the effect of NO on the symbiotic phenotype differs depending on the timing and concentration of NO production and on whether host plant nodules are determinate or indeterminate. *Medicago truncatula* forms indeterminate nodules, and NO is necessary for optimal symbiosis with its microsymbiont *Sinorhizobium meliloti* [25]. In contrast, *Lotus japonicus* forms determinate nodules, and NO levels that are too high can inhibit infection and nodulation [26]. Regardless of nodule type, NO inhibits nitrogenase activity, such that decreased levels of NO in the nodules contribute to higher nitrogenase activity and delayed nodule senescence [26,27,28,29,30,31]. Generally, infection with symbiotic microbes suppresses the expression of pathogenesis-related genes; in *M. truncatula,* ROS are necessary for symbiosis because of their function as negative regulators of pathogenesis-related genes [32]. The role of ROS in symbiosis between *Phaseolus vulgaris* and *Rhizobium tropici* has also been reported [33]. In *P. vulgaris,* the NADPH oxidase-encoding gene *PvRbohA* is responsible for ROS production. In *PvRbohA*-RNAi lines, PvRbohA-dependent ROS production is attenuated in rhizobia-inoculated roots. Furthermore, the rhizobial infection events are significantly reduced in *PvRbohA*-RNAi lines, resulting in ineffective nodule formation. Considering that symbiotic rhizobia defective in ROS elimination show reduced symbiotic nitrogen fixation activity [34], an unknown mechanism must operate to keep ROS levels in nodules at suitable levels.

In both animals and plants, most H_2_S signaling relates to or is mediated by NO or ROS [14,35,36,37]. However, the target molecules and mechanism of H_2_S have yet to be clarified. Zou et al. [38] reported that providing an H_2_S donor promoted nodulation and symbiotic nitrogen fixation in soybean, suggesting that H_2_S positively participates in root nodule symbiosis.

Reactive sulfur species (RSS), such as polysulfides, which interact with NO, ROS, and H_2_S in animals and bacteria, are garnering increasing interest [39,40]. RSS are characterized as redox-active sulfur-containing molecules that are able, under physiological conditions, to either oxidize or reduce biomolecules [39]. H_2_S can also be classified as an RSS because it enters into redox reactions with proteins and affects their activities [39]. However, in this study, H_2_S was considered as a signaling molecule, and RSS were defined at polysulfides detected using SSP4. Multivalent sulfur molecules such as polysulfides [R-S-(S)_n_H] can sulfurize cysteine, glutathione, and intra-protein thiols (SH) to produce persulfides (R-S-SH), which are involved in signal transduction and redox control [41,42,43]. RSS have higher nucleophilicity than conventional thiol compounds and also have strong antioxidant activity [42,43]. The production and function of RSS have been analyzed since the 1960s, mainly in animals and bacteria [44,45,46,47,48,49,50]. RSS interact with NO, ROS, and H_2_S and are expected to function collaboratively with these molecules in plants as well as in animals and bacteria [39,40]. Although the production and function of RSS in plants have been discussed [51,52,53], few reports based on experimental evidence have been published.

The purpose of the present study was to investigate whether RSS was involved in root nodule symbiosis. In the present study, polysulfides were detected in the roots of the model legume *L. japonicus* by using a sulfane sulfur–specific fluorescent probe, SSP4. Supply of exogenous Na_2_S_3_ as a sulfane sulfur donor affected the amount of ROS, NO, and H_2_S in the roots and promoted infection thread (IT) formation, suggesting that polysulfides might positively regulate the infection process via its effects on ROS, NO, and H_2_S. Although polysulfides were detected in the nodules, supply of exogenous Na_2_S_3_ to nodules decreased nitrogen fixation activity, suggesting that the RSS concentration is precisely controlled in nodules. RSS may function collaboratively with ROS, NO, and H_2_S in the legume–rhizobium symbiosis.

## 2. Materials and Methods 

### 2.1. Biological Materials and Growth Conditions

Seeds of *Lotus japonicus,* accessions MG-20 and Gifu B-129, were germinated and grown as described previously [54]. In brief, three days after germination of MG-20, seedlings were transferred to 1.5% Fåhraeus agar plates [55]. For observation of IT development and nodulation, seedlings of Gifu B-129 were transferred to pots filled with vermiculite moistened with Fåhraeus liquid medium 5 days after germination. Each seedling was inoculated with a cell suspension (10^7^ cells ml^–1^ in water) of *Mesorhizobium loti* MAFF303099 [56] or MAFF303099 DsRed [57]. The number of ITs and nodules were counted at 10 days post inoculation (dpi) and at one month post inoculation (mpi). The plants were grown under controlled conditions with a photosynthetically active radiation of 150 μmol photons m^–2^ s^–1^ (16-h photoperiod) at 25 °C.

### 2.2. Nitrogenase Activity

Nitrogenase activity of nodules was determined as acetylene reduction activity, according to Shimoda et al. [28]. Whole plants were placed in glass tubes containing wet filter paper. The tubes were filled with an acetylene and air mixture (C_2_H_2_: air = 1:9 v/v). After 2 h of incubation at 25 °C, the amount of ethylene in the gas phase was determined using a GC-3A gas chromatograph (Shimadzu, Kyoto, Japan).

### 2.3. Endogenous Signal Molecule Production in Roots and Nodules

Roots and agar sections of nodules were soaked for 1 h with specific probes for each molecule, as follows. The probes were dissolved in phosphate-buffered saline; 137 mM NaCl, 2.7 mM KCl, 8 mM Na_2_HPO_4_, 2 mM NaH_2_PO_4_ (pH 7.4). For polysulfide detection, roots and nodules were soaked in 10 μM SSP4 (Dojindo, Kumamoto, Japan) with 0.5 mM cetyltrimethylammonium bromide. SSP4 has no membrane permeability, but can penetrate cell membranes by combination with cetyltrimethylammonium bromide. SSP4 reacts with sulfen sulfur as well as with polysulfides of glutathione and of cysteine. For NO detection, the samples were soaked in 20 μM DAF-FM DA (GORYO Chemical, Sapporo, Japan). DAF-FM DA has membrane permeability and is deacetylated to DAF-FM by esterase inside the cell, where DAF-FM reacts with the endogenous NO oxidation product N_2_O_3_ to form a highly fluorescent triazole. For ROS detection, the samples were soaked in 10 μM CellROX Green Reagent (Invitrogen, Carlsbad, CA, USA). The cell-permeable CellROX reagent is essentially non-fluorescent while in a reduced state and exhibits a strong fluorescent signal upon oxidation. For H_2_S detection, the samples were soaked in 5 μM Hsip-1 DA (Dojindo). Hsip-1 DA has membrane permeability. By esterase activity inside the cell, Hsip-1 DA is deacetylated to Hsip-1, which is a fluorescent molecule of the copper (II) ion chelate type and reacts specifically with hydrogen sulfide. Nodule cell walls were stained with calcofluor white stain (Sigma-Aldrich, Oakville, ON, Canada). Infected cells in nodule tissue were stained by soaking in 10 μM 4’,6-diamidino-2-phenylindole (Dojindo). Endogenous production of all signal molecules in the roots was observed by fluorescence microscopy or stereofluorescence microscopy. Confocal images were captured using an A1si-90i microscope (Nikon, Tokyo, Japan) and epifluorescence images using an Eclipse 90i microscope (Nikon). The fluorescence intensity was quantified by using the image analysis software Image J (Version 1.51, NIH, Bethesda, MD, USA).

### 2.4. Treatment of Roots and Nodules with the Sulfane Sulfur Donor Na_2_S_3_

Na_2_S_3_ (Dojindo) solution was prepared just before use according to the manufacturer’s protocol. To investigate the effect of RSS on the quantities of endogenous signal molecules, seedlings and agar sections of nodules were soaked in 1 mM Na_2_S_3_ for 1 h, and washed twice with phosphate buffer, then labeled with each fluorescent probe. To investigate the effect of RSS on nitrogenase activity, roots with nodules at 4 wpi were soaked in 100 μM, 500 μM, or 1 mM Na_2_S_3_ for 12 h. To investigate the effect of RSS on IT formation and nodulation, inoculated seedlings were treated with 100 μL of 500 μM Na_2_S_3_ at 72 h intervals for 10 days because Na_2_S_3_ is not stable in the physiological solution. 

## 3. Results

### 3.1. Detection of Polysulfides in the Roots of Lotus japonicus by using SSP4

**Figure 1:**
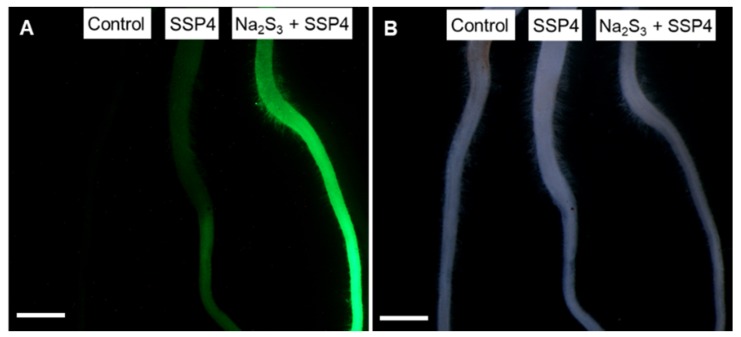
Detection of reactive sulfur species (RSS) in the roots of *Lotus japonicus* MG-20. Seedlings were incubated for 1 h with SSP4 for detection of RSS or incubated with the sulfane sulfur donor Na_2_S_3_ for 1 h prior to incubation with SSP4. Fluorescence (**A**) and bright field (**B**) images. Control seedlings were incubated with phosphate-buffered saline. Scale bars, 5 mm.

Seedlings of *L. japonicus* were treated with SSP4 for 1 h prior to observation with a stereo fluorescence microscope. Roots treated with SSP4 showed stronger fluorescence intensity than untreated roots (Figure 1A). Roots treated with the sulfane sulfur donor (Na_2_S_3_) for 1 h before SSP4 treatment showed stronger fluorescence intensity than those not treated with the donor (Figure 1A). Thus, plant RSS were detectable with the polysulfide-specific fluorescent probe SSP4. The roots were treated with various concentrations of the donor and the fluorescence intensity of SSP4 was observed by fluorescence microscope. The Na_2_S_3_ significantly increased the fluorescence intensity of SSP4 in a dose-dependent manner (Supplementary Figure S1) and the intensity of the root without Na_2_S_3_ treatment appeared to be lower than that of treated with 10 μM of Na_2_S_3_, suggesting that the endogenous RSS in *L. japonicus* roots was less than 10 μM.

### 3.2. Effect of Na_2_S_3_ on the Amount of NO, ROS, and H_2_S in Roots

**Figure 2:**
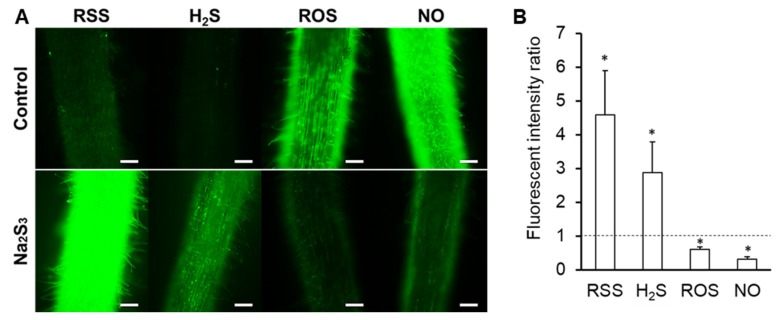
Effect of Na_2_S_3_ on the amount of nitric oxide (NO), reactive oxygen species (ROS), and hydrogen sulfide (H_2_S) in roots of *L. japonicus* MG-20. (**A**) Fluorescence microscopy images of root tissue, showing signal molecules. Seedlings were incubated with Na_2_S_3_ for 1 h prior to incubation with each specific probe. Scale bars, 100 μm. (**B**) Fluorescence intensity was quantified by using the image analysis software Image J. Fluorescence intensity of Na_2_S_3_-treated roots is expressed relative to that of untreated roots (control) which was set at 1, indicated by the dashed line. Values indicate means (± SE; *n* = 30). Asterisks denote statistically significant differences compared to the control (Student’s *t*-test, * *p* < 0.05).

To investigate the relationships among RSS, NO, ROS, and H_2_S in plants, seedlings were treated with Na_2_S_3_ for 1 h and then fluorescent probes specific to each molecule (SSP4, DAF-FM DA, CellROX, Hsip-1 DA) were used. Roots treated with Na_2_S_3_ showed stronger SSP4 fluorescence, indicating greater polysulfide content than untreated roots, as expected (Figure 2A). In roots treated with Na_2_S_3_, the intensities of DAF-FM DA fluorescence and CellROX fluorescence were lower than in untreated roots, whereas the intensity of Hsip-1 DA fluorescence was higher than in untreated roots (Figure 2A). These results indicate that the sulfane sulfur donor reduced NO and ROS and increased H_2_S in the roots. Analyses using Image J showed that these differences in fluorescence intensity were significant (Figure 2B).

### 3.3. Localization of RSS and Signal Molecules during Rhizobial Infection

**Figure 3:**
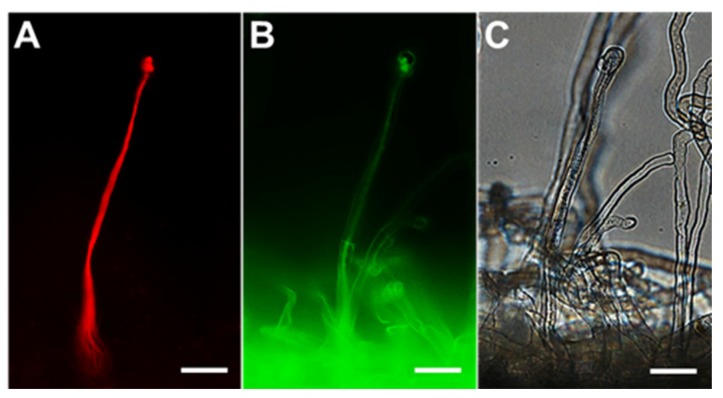
Detection of reactive sulfur species during infection. (**A**) Infection thread produced by *Mesorhizobium loti* MAFF303099 DsRed. (**B**) Polysulfide detected by using the specific probe SSP4. (**C**) Bright field observation. *L. japonicus* B-129 was used as a host plant. Scale bars, 25 μm.

**Figure 4:**
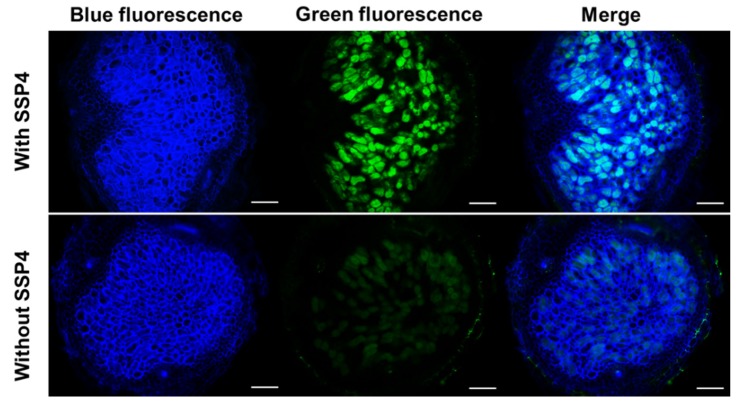
Detection of reactive sulfur species in nodules. Blue fluorescence indicates nodule cell walls stained with calcofluor white. Green fluorescence indicates RSS labeled with SSP4. The green fluorescence of without SSP4 shows autofluorescence. *L. japonicus* B-129 was used as a host plant. Scale bars, 100 μm.

To investigate the involvement of RSS in root nodule symbiosis, localization of polysulfides during the rhizobial infection process was observed by using SSP4. Ten days after inoculation with DsRed-labeled *Mesorhizobium loti*, polysulfides in the roots of *L. japonicus* were observed by using a fluorescence microscope and the SSP4 probe. The ITs could be clearly observed in the root hairs (Figure 3A,C), and polysulfides were found to overlap with ITs (Figure 3B). Localization of NO, ROS, and H_2_S was also examined using specific fluorescent probes. NO and ROS were detected only in a limited area, including the tip and base of the ITs (), whereas H_2_S was not detected in ITs (Supplementary Figure S2B). Localization of polysulfides, NO, ROS, and H_2_S in root nodules, which are symbiotic organs, was also examined. Agar sections of mature nodules were treated with each specific fluorescent probe and observed. Polysulfides were detectable in infected cells (Figure 4), as were the three other molecules (Supplementary Figure S3).

### 3.4. Effect of RSS on Rhizobial Infection

**Figure 5:**
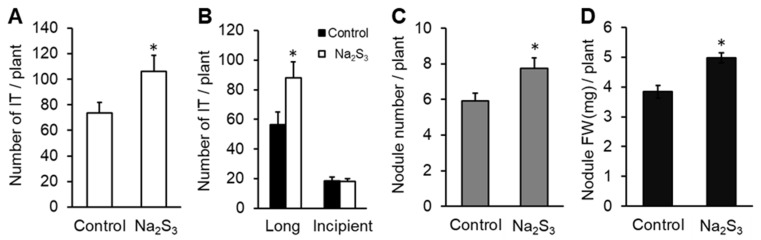
Effect of Na_2_S_3_ on infection threads (ITs) and nodules. Na_2_S_3_ was added every 72 h during the first 10 days after inoculation of *Mesorhizobium* *loti*. (**A**) Total number of ITs. (**B**) Numbers of long or incipient ITs counted at 10 dpi. (**C**) Nodule number and (**D**) nodule fresh weight (mg) per plant were measured at 1 month post inoculation (mpi). *L. japonicus* B-129 was used as a host plant. Values are shown as means ± SE; *n* = 24 for number of infection threads and *n* = 18 for number and fresh weight of nodules. Asterisks denote statistically significant differences compared to controls (Student’s *t*-test, * *p* < 0.05).

Na_2_S_3_ was applied to *L. japonicus* seedlings, and the number of ITs was counted at 10 days after inoculation of *M. loti*. ITs were counted in two groups (incipient and long ITs) according to the terminology of Małolepszy et al. [58], except that in our case the elongating ITs were also considered as long ITs. The total number of ITs was significantly higher in roots treated with the donor than in control roots (Figure 5A). The number of long ITs was significantly higher in roots treated with the donor than in control roots, although no difference was observed in the number of incipient ITs (Figure 5B). At 1 month after inoculation, the number and fresh weight of nodules on roots treated with the donor was significantly higher than on control roots (Figure 5C,D).

### 3.5. Effects of RSS on Nitrogenase Activity of Nodules

**Figure 6:**
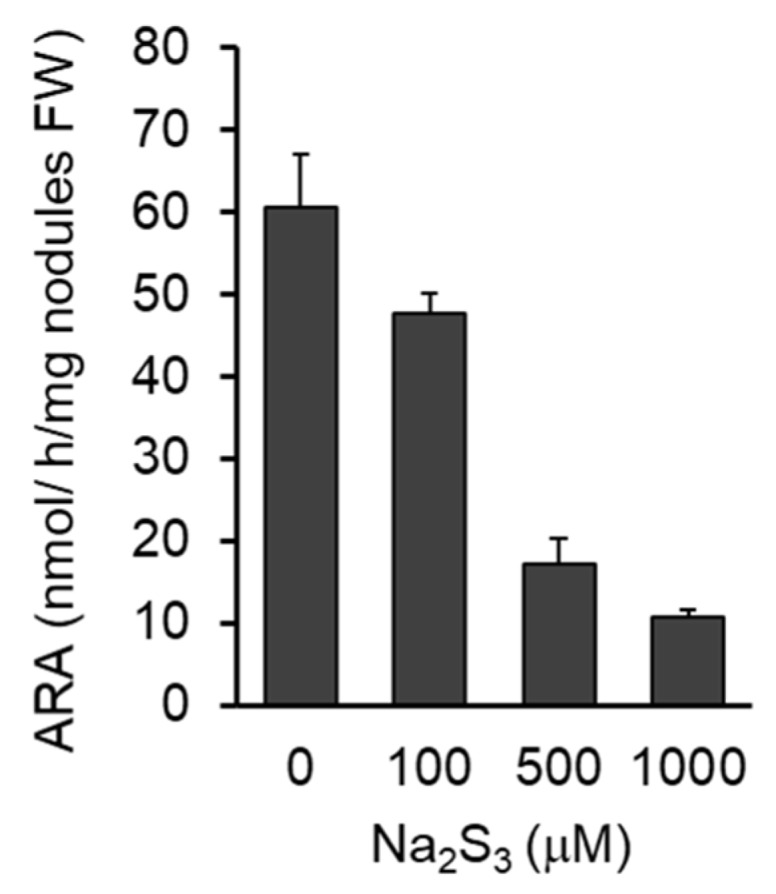
Effect of Na_2_S_3_ on nitrogenase activity of nodules. Acetylene reduction activity (ARA) was measured at 4 weeks post inoculation. *L. japonicus* MG-20 was used as a host plant. Values indicate means ± SE (*n* = 12). All values showed significant difference (Student’s *t*-test, *p* < 0.05).

Because RSS were detected in infected cells in the nodules, the effect of exogenous Na_2_S_3_ on the nitrogenase activity of the nodules was investigated. Four weeks after inoculation, the nodules were treated with various concentrations of the donor for 12 h and the nitrogenase activity was measured. The sulfane sulfur donor significantly decreased the acetylene reduction activity of the nodules in a dose-dependent manner (Figure 6).

### 3.6. Effects of RSS on Signal Molecules in Infected Cells

**Figure 7:**
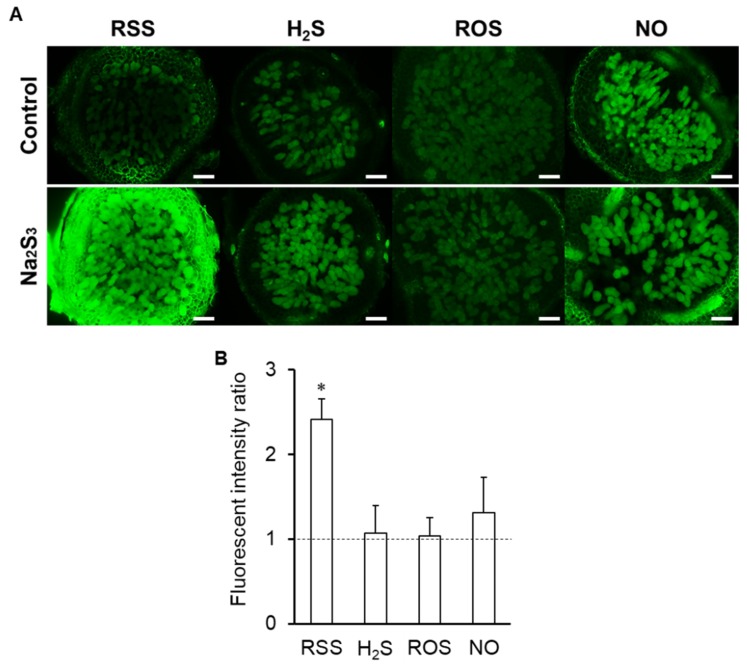
Effect of Na_2_S_3_ on quantity of reactive sulfur species (RSS), nitric oxide (NO), reactive oxygen species (ROS), and hydrogen sulfide (H_2_S) in infected cells. (**A**) Fluorescence microscopy images of agar sections of nodules incubated with Na_2_S_3_ prior to incubation with each specific fluorescent probe. Scale bars, 100 μm. (**B**) Fluorescence intensity was quantified by using Image J. Fluorescence intensity of Na_2_S_3_-treated roots is expressed relative to that of untreated roots (control) which was set at 1, indicated by the dashed line. Values indicate means (± SE; n=18). Asterisks denote statistically significant differences compared to the control (Student’s *t*-test, * *p* < 0.05). *L. japonicus* MG-20 was used as a host plant.

Because RSS localized in the infected cells of nodules, the effect of RSS on signal molecules in the infected cells was examined by each specific fluorescent probe after nodule sections were treated with Na_2_S_3_. Exogenous Na_2_S_3_ significantly increased SSP4 fluorescence intensity, as expected (Figure 7). On the other hand, fluorescence of NO, ROS, and H_2_S showed no significant changes compared to controls (Figure 7A), as supported by image analysis (Figure 7B).

## 4. Discussion

Signaling cross-talk between NO, ROS, and RSS (including H_2_S) and its potential functions has been reported in plants [51,52,53,59]. In the present study, we provide evidence that (1) RSS are detectable in the roots of *Lotus japonicus* by using a specific fluorescent probe, (2) RSS are involved in *Mesorhizobium–Lotus* symbiosis, and (3) they collaborate with these signal molecules.

Exogenous Na_2_S_3_ affected concentrations of NO, ROS, and H_2_S in *L. japonicus*, as reported in animals and bacteria [60,61]. H_2_S is a by-product of the metabolic pathways in animals and bacteria that produce other RSS [45,46,47,48,49,50]. In *L. japonicus*, the amount of H_2_S in the roots was significantly increased by exogenous Na_2_S_3_ (Figure 2A,B), suggesting that plants possess a RSS metabolic pathway similar to those of animals and bacteria, and that they may have similar functions and roles in vivo. Exogenous Na_2_S_3_ significantly decreased ROS in roots (Figure 2). Given that the mechanism for removal of ROS by RSS has been reported in animals and bacteria [42,62], a mechanism for indirect and/or direct ROS elimination may also be present in plants. NO was also significantly decreased in the roots by exogenous Na_2_S_3_ (Figure 2). It is not clear whether RSS eliminate NO or inhibit NO production, as data on this phenomenon are lacking. Exogenous Na_2_S_3_ decreased NO as well as ROS, and it would be worth investigating whether this occurs not only in plants but also in animals and bacteria. Because providing the sulfane sulfur donor Na_2_S_3_ increased H_2_S and decreased ROS in roots (Figure 2), we must carefully consider not only the direct relationship between RSS and NO, but also interactions with H_2_S and ROS. In addition, because Na_2_S_3_ is not stable in physiological solution, new reactive species might be formed after several seconds-minutes of the incubation as previous report [63]. Interaction among the new reactive species, ROS, NO, and H_2_S, should be also considered.

NO, ROS, and H_2_S are involved in root nodule symbiosis [24,38,64]. Because RSS interact with these signal molecules, we investigated RSS in root nodule symbiosis. Polysulfide localized at the ITs (Figure 3) and Na_2_S_3_ significantly promoted IT formation and nodulation (Figure 5). These results suggest that RSS are involved in the infection and nodulation process in *L. japonicus–M. loti* symbiosis. In *L. japonicus*, NO inhibits formation of ITs and nodulation [27]. Thus, exogenous Na_2_S_3_ might promote IT formation and nodulation by decreasing NO in roots. ROS, which decreased by Na_2_S_3_, could affect the infection and nodulation of *L. japonicus* because ROS are also key players in bacteria–plant interactions and IT formation [24,34,64,65]. ROS regulation by microsymbionts should also be considered because two important ROS-scavenging enzymes, superoxide dismutase and catalase, are essential for the establishment and maintenance of symbiosis between alfalfa and *S. meliloti* [66,67]. Recently, Zou et al. [38] reported that addition of a H_2_S donor promoted IT formation and nodulation in soybean. In the present study on *L. japonicus*, the increase in H_2_S promoted by RSS might also promote infection and nodulation (Figure 2, Figure 5).

RSS were also detected in infected cells in nodules (Figure 4), and exogenous Na_2_S_3_ significantly decreased nitrogenase activity in nodules (Figure 6). These results suggest that RSS are involved not only in the infection process but in symbiotic nitrogen fixation as well. There are several possible explanations for the decreased nitrogenase activity caused by the exogenous Na_2_S_3_. RSS could be inhibitors of nitrogenase or affect the activities of molecules that are essential for symbiosis. Several symbiosis-related molecules, including leghemoglobin and glutamine synthetase, are post-translationally modified, for example nitration via NO and ROS during symbiosis [68,69,70]. RSS are known to be involved in post-translational modification of proteins [71], and excess RSS in infected cells might cause abnormal modification of symbiosis-related proteins. We do not exclude the possibility that RSS affect the biological activity of bacteroids in infected cells because bacteroids have highly permeable cell membranes [72,73] and are susceptible to their external environment.

Exogenous Na_2_S_3_ did not affect the amount of NO, ROS, or H_2_S in nodules, whereas Na_2_S_3_ significantly decreased NO and ROS and increased H_2_S in roots (Figure 2, Figure 7). These results suggest differences in metabolism and response between nodules and roots, although the membrane permeability of each fluorescent probe should be considered in the different tissues. Na_2_S_3_ decreased nitrogenase activity, whereas RSS were detected in infected cells, suggesting that the amount of RSS in the infected cells is regulated for nitrogenase activity. Despite being inhibitors of nitrogenase [26,34,66,67,74,75], NO and ROS are necessary for establishment of proper symbiosis [23,64]. In this context, RSS, like NO and ROS, could be a double-edged sword for symbiosis. A sulfate transporter SST1 is essential for symbiotic nitrogen fixation in *L. japonicus* [76]. Considering the importance of sulfur transport and metabolism in the root nodule symbiosis as recently reviewed [77], RSS will be one of the important players in the symbiosis. Although our experimental results suggest that RSS is involved in the symbiosis, it is necessary to investigate the RSS concentration throughout the symbiotic process and to reconsider the effect of RSS on the symbiosis. Both host plant and rhizobial mutants on RSS production will allow us to understand the function of RSS and provide new insights into sulfur metabolism and RSS in the root nodule symbiosis.

## 5. Conclusion

In the present study, RSS were detected in *L. japonicus*, and cross-talk with three signal molecules, NO, ROS, and H_2_S, was suggested. These signal molecules function in various important physiological processes in plants, such as germination, opening and closing of stomatal guard cells, and photosynthesis, as well as in abiotic and biotic stress responses including plant–microbe interactions. However, these processes are not yet completely understood. RSS may be a new player, collaborating with NO, ROS, and H_2_S in these processes, and studying RSS will help us to understand the diverse physiological processes of plants.

## Supplementary Materials

The following are available online at https://www.mdpi.com/2076-3921/9/2/145/s1, Figure S1; Detection of reactive sulfur species in the roots of *Lotus japonicus* treated with various concentration of Na_2_S_3_. (A) Seedlings were incubated with the 1, 10, 100 µM and 1 mM of Na_2_S_3_ for 1 h prior to incubation with SSP4 for 1 h. Without SSP4, seedlings were incubated with phosphate-buffered saline alone for 2 h. + and - indicate the presence or absence of SSP4 or Na_2_S_3_. Scale bars, 100 μm. (B) Fluorescence intensity was quantified by using Image J. Fluorescence intensity of Na_2_S_3_-treated roots is expressed relative to that of without Na_2_S_3_ which was set at 1. Values indicate means (± SE; n=18). Means denoted by the same letter do not differ significantly by Student’s *t*-test at *P* < 0.05. Figure S2; Detection of reactive sulfur species (RSS), hydrogen sulfide (H_2_S), reactive oxygen species (ROS), and nitric oxide (NO) during infection by fluorescence microscopy. (A), polysulfides; (B), H_2_S; (C), ROS; (D), NO. Polysulfide, H_2_S, ROS, and NO were detected as green fluorescence produced by the specific probes SSP4, Hsip-1 DA, CellROX, and DAF-FM DA, respectively. Red fluorescence indicates the infection thread produced by *Mesorhizobium loti* MAFF303099 DsRed in A–D. *L. japonicus* B-129 was used as a host plant. Scale bars, 25 μm. Figure S3; Localization of reactive sulfur species (RSS), hydrogen sulfide (H_2_S), reactive oxygen species (ROS), and nitric oxide (NO) in nodules of *L. japonicus* MG-20. Green fluorescence indicates polysulfides, H_2_S, ROS, and NO in the nodule detected by each specific probe. The green fluorescence overlaps with the blue fluorescence of infected cells stained with 4’,6-diamidino-2-phenylindole. Scale bars, 100 μm.
