# Supplementary figures and images for: Reactive Sulfur Species Interact with Other Signal Molecules in Root Nodule Symbiosis in Lotus japonicus
Source: Antioxidants (Basel). 2020 Feb 7;9(2):145. doi: 10.3390/antiox9020145 (PMC7070391; doi:10.3390/antiox9020145)

# Supplemental Figure S1

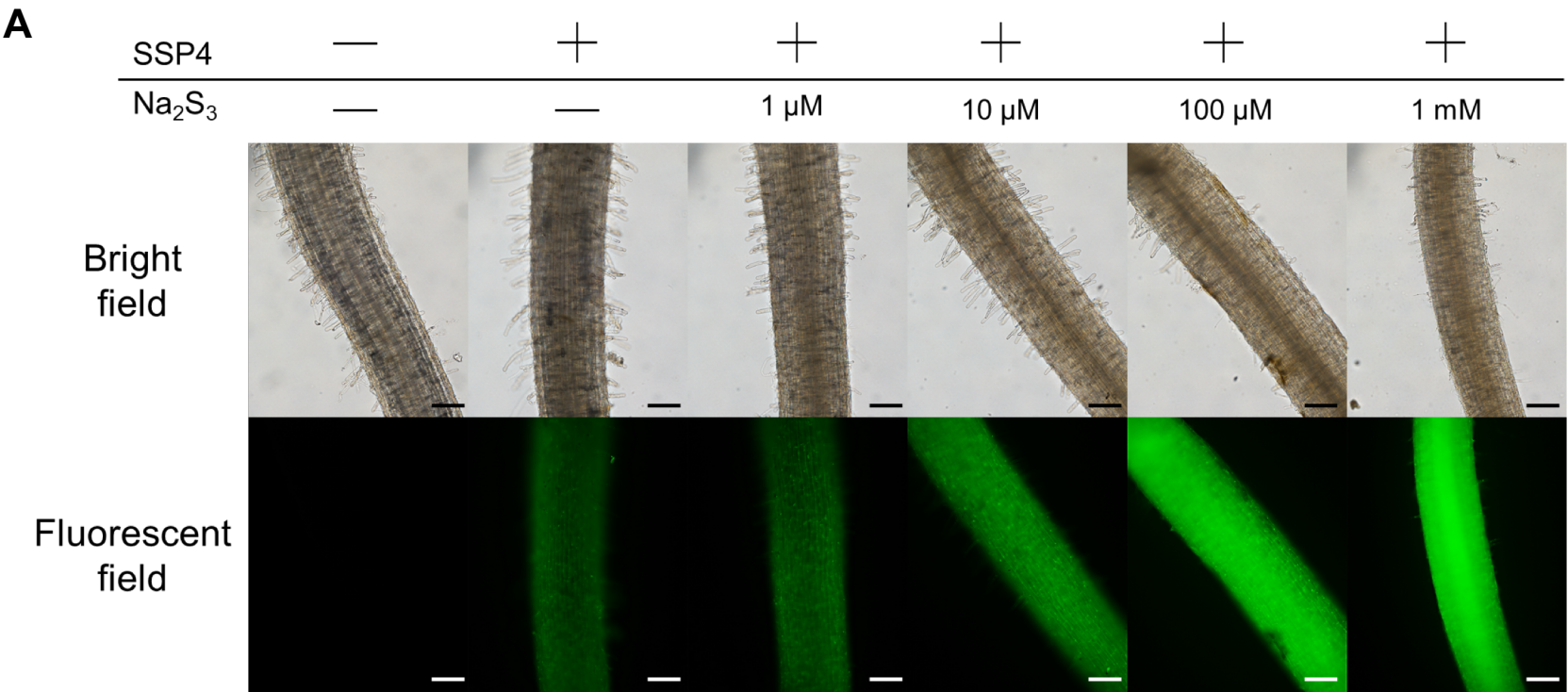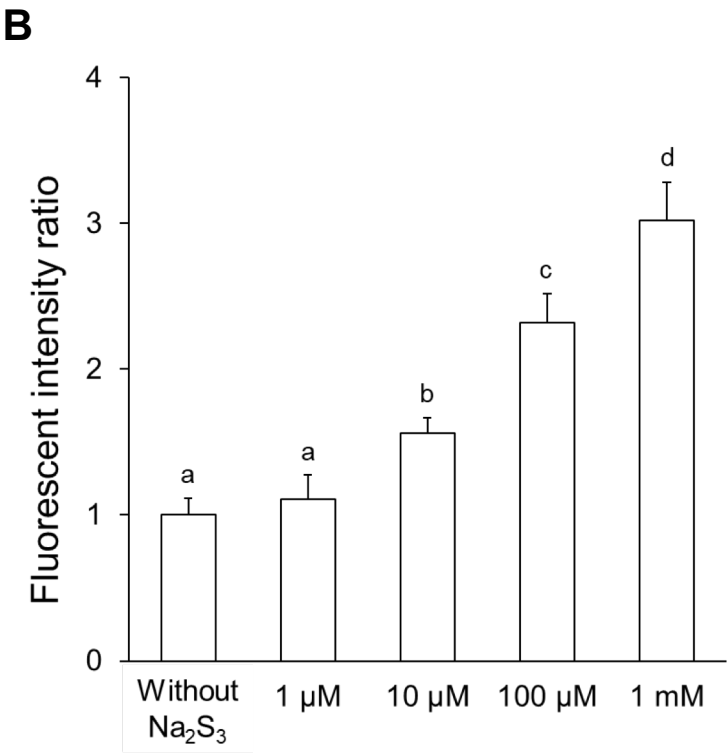

# Supplemental Figure S2

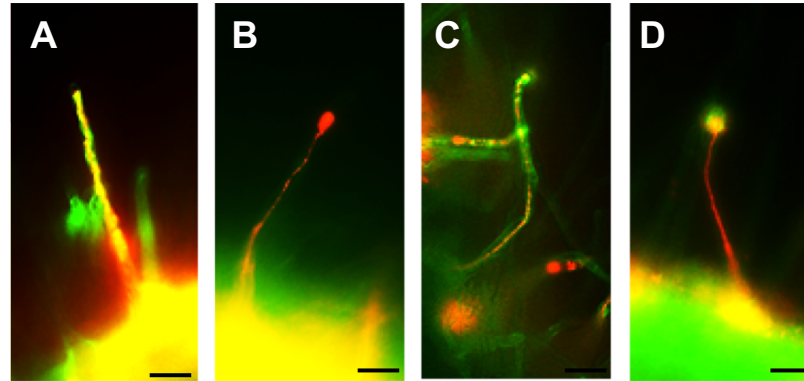

# Supplemental Figure S3

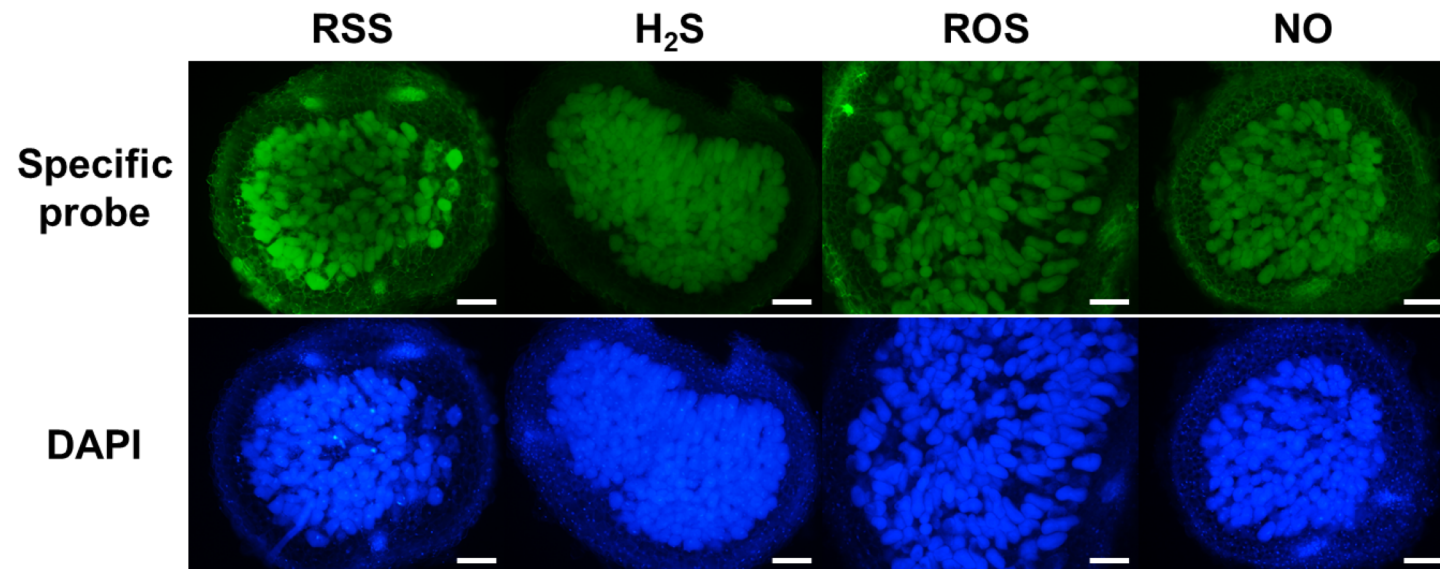

Supplement: Supplementary file 1 [file antioxidants-09-00145-s001.pdf]
